# Combined Endovascular and Microsurgical Management of Complex Cerebral Aneurysms

**DOI:** 10.3389/fneur.2013.00108

**Published:** 2013-08-08

**Authors:** Omar Choudhri, Nitin Mukerji, Gary K. Steinberg

**Affiliations:** ^1^Department of Neurosurgery, Stanford Stroke Center, Stanford Institute for Neuro-Innovation and Translational Neurosciences, Stanford University School of Medicine, Stanford, CA, USA

**Keywords:** coil embolization, combined therapy, complex aneurysms, microsurgical clipping, revascularization bypass, vessel sacrifice

## Abstract

Cerebral aneurysms are associated with a 50% mortality rate after rupture and patients can suffer significant morbidity during subsequent treatment. Neurosurgical management of both ruptured and unruptured aneurysms has evolved over the years. The historical practice of using microsurgical clipping to treat aneurysms has benefited in the last two decades from tremendous improvement in endovascular technology. Microsurgery and endovascular therapies are often viewed as competing treatments but it is important to recognize their individual limitations. Some aneurysms are considered complex, due to several factors such as aneurysm anatomy and a patient’s clinical condition. A complex aneurysm often cannot be completely excluded with a single approach and its successful treatment requires a combination of microsurgical and endovascular techniques. Planning such an approach relies on understanding aneurysm anatomy and thus should routinely include 3D angiographic imaging. In patients with ruptured aneurysms, endovascular coiling is a well-tolerated early treatment and residual aneurysms can be treated with intervals of definitive clipping. Microsurgical clipping also can be used to reconstruct the neck of a complex aneurysm, allowing successful placement of coils across a narrow neck. Endovascular techniques are assisted by balloons, which can be used in coiling and testing parent vessel occlusion before sacrifice. In some cases microsurgical bypasses can provide alternate flow for planned vessel sacrifice. We present current paradigms for combining endovascular and microsurgical approaches to treat complex aneurysms and share our experience in 67 such cases. A dual microsurgical–endovascular approach addresses the challenge of intracranial aneurysms. This combination can be performed safely and produces excellent rates of aneurysm obliteration. Hybrid angiographic operating-room suites can foster seamless and efficient *complementary* application of these two modalities.

## Introduction

Cerebral aneurysms are the leading cause of non-traumatic subarachnoid hemorrhage (SAH) and account for 70–80% of SAH cases (1). Untreated ruptured cerebral aneurysms are associated with a high mortality and a risk of rebleeding (2). A large part of cerebrovascular neurosurgery is directed toward diagnosis and treatment of aneurysms before they rupture. The rupture risk of cerebral aneurysms is directly related to the size, shape, and location of the aneurysm based on large center studies (3, 4).

Treatment of intracranial aneurysms has evolved appreciably in the past 100 years with improvements in diagnosis and surgical techniques (5). Microsurgical clipping and endovascular coil embolization are the two main treatment strategies for obliteration of ruptured and unruptured aneurysms.

Aneurysms traditionally have been treated with a craniotomy and microsurgical clipping across the neck of the aneurysm (6). Craniotomy and clipping of aneurysms is a moderate risk surgery that is tolerated fairly well, depending on the pre-treatment clinical grade of the patient.

Historically the treatment of cerebral aneurysms involved ligation of proximal parent vessel known as Hunterian ligation. Victor Horsely (1857–1916) first used this technique to treat a giant internal carotid artery (ICA) aneurysm in 1885. Norman Dott (1897–1973) and Harvey Cushing (1869–1939) subsequently introduced muscle wrapping and suture ligation for intracranial aneurysms. Harvey Cushing and Walter Dandy (1886–1946) are credited with introducing microsurgical clips to treat intracranial aneurysms. (7) In 1937 Dandy was the first to use a V-shaped silver clip to perform the first clipping of an ICA aneurysm (5). Since the time of Cushing and Dandy aneurysm clipping has come a long way with improvements to clip design, neurosurgical microscopes, vascular imaging, intraoperative monitoring, and neuroanesthesia. Most series report an aneurysm exclusion rate of 92–96% with microsurgical clipping (8).

While microsurgery for intracranial aneurysms has evolved over the past century, endovascular therapy has also progressed in parallel. Endovascular therapy obliterates an aneurysm by occluding its dome with thrombogenic coils. Electrothermal coagulation of aneurysms was attempted as early as the 1940s. From the 1960s to the 1980s detachable balloon catheters were used to obliterate intracranial aneurysms with suboptimal results (9, 10). This led to the development of pushable platinum coils, which were fraught with coil retrieval and coil migration issues (9). Guido Guglielmi, an Italian neurosurgeon, was the first to introduce the modern detachable platinum coil – the Guglielmi detachable coil or GDC (11). The first aneurysm treated with a detachable platinum coil where the coil position could be examined before detachment occurred in 1990. This technology allowed controlled coil embolization of an aneurysm and started the modern era of endovascular aneurysm treatment. GDCs were approved by the FDA for use with intracranial aneurysms in 1995, recognizing coiling as a competitive treatment strategy. Balloon-assisted coiling, stent-assisted coiling, and flow diverter technology have been subsequently introduced with improved aneurysm occlusion rates by way of aneurysm remodeling (10). Neuroradiologists, neurosurgeons, and neurologists have been active in this field for the past two decades and endovascular technology has advanced immensely. These advances include improved biplane flat panel imaging, catheter and microcatheter development, softer detachable coils, compliant intracranial balloons, intracranial stent development, and flow diverters (12). Complete exclusion rates with endovascular coiling are reported as 40–55% while the near complete exclusion rates are an additional 35.4–52% (8). Given improvements in both microsurgical clipping and endovascular therapy, both therapies are effective if used in the correct clinical setting.

Which treatment modality is best in terms of aneurysm protection and durability along with minimal recurrence and morbidity is, however, a widely debated topic. The International Subarachnoid Aneurysm Trial (ISAT) and Barrow Ruptured Aneurysm Trial (BRAT) compared endovascular coiling to microsurgical clipping in ruptured aneurysms amenable to both treatments (13, 14). These studies concluded that outcomes at 1 year for coil embolization were better for endovascular coiling than clipping. The 3-year results of BRAT indicated that the clipping group had more aneurysm obliteration and less recurrence. No significant difference in outcomes was noted between the two modalities, especially for anterior circulation aneurysms (15). Microsurgical clipping was the standard of care for aneurysm treatment before 1995. Based on a national inpatient sample (16) from 2002 to 2008, a majority of patients have undergone endovascular coil embolization for ruptured and unruptured aneurysms (16). There is a variation in adoption of endovascular treatment across different hospitals. A higher percentage of endovascular procedures for aneurysms are completed at urban academic health centers (17).

The decision to triage patients for surgery and/or endovascular treatment benefits from a multidisciplinary approach to optimal aneurysm protection and patient outcome. For most aneurysms a single well-chosen treatment modality has clear advantages and can ensure adequate aneurysm protection. This is less straightforward for a subgroup of complex aneurysms though, which may require multimodal treatment. Hacein-Bey et al. describe features of complex aneurysms as a combination of anatomical aneurysm factors and clinical factors (Table 1) (18). In the present reviewers’ experience microsurgical and endovascular treatments can be complementary in the management of complex cerebral aneurysms.

**Table 1:** Features contributing to complex aneurysms*.

Aneurysm anatomy (best assessed by 3D aneurysm reconstruction from DSA or CTA data)	Clinical features (detailed clinical risk stratification important)
**Size:** large or giant, too small for a clip or coil	**Clinical grade at presentation:** HH ≥3
**Shape:** fusiform, serpentine, pseudoaneurysm, dissecting aneurysm	**Timing:** vasospasm at the time of presentation
**Content:** filled with thrombus, calcified wall, dysplastic vessel wall	**Medical comorbidities:** cardiovascular, pulmonary, renal or endocrine comorbidity
**Neck:** difficult surgical access, broad, calcified, involving perforator vessels, and other branching vessels	**Advanced age**
**Perianeurysmal environment:** aneurysm embedded in eloquent brain tissue, bone, edema, scar from previous surgery	

*DSA, digital subtraction angiography; CTA, computed tomography angiography; HH, Hunt and Hess*.

*Used Table from (18) with permission from Wolters Kluwer Health with modification.

This review highlights the limitations of each treatment modality and how those limitations can be overcome by astutely combining microsurgery and endovascular coiling. Combined treatment of complex aneurysms was first reported by our center at Stanford University, followed by several others with similar experiences (19–25). Here, we discuss our experience thus far in the combined treatment of cerebral aneurysms and describe a number of cases. These combined treatments often follow the failure of an earlier therapy, but can also be used adjunctively in a planned fashion.

## Microsurgical Clipping and Its Limitations

Microsurgical clipping requires the completion of a precise skull-base approach that provides safe access to the aneurysm and allows a microsurgical clip to be placed across its neck. A successful operation depends upon a carefully thought out plan comprising craniotomy, skull-base drilling, dural exposure, arachnoid dissection and, ultimately, dissection of the aneurysm and its surrounding blood vessels. These steps require microsurgical finesse and are challenging in patients with ruptured aneurysms and cerebral edema. Appropriate brain relaxation is crucial and attempts are made to minimize brain retraction while approaching the circle of Willis at the skull base. Additionally, recognition of normal vascular anatomy and perforator vessels are important to prevent infarcts during clipping. (5) Often clip reconstruction and a combination of clips are used to protect vessels originating from the aneurysm.

Aneurysm morphology is important to determine before deciding between the treatments. A 3D reconstruction of a cerebral angiogram can provide an accurate estimate of aneurysm size and morphology. Microsurgical treatment requires a craniotomy and therefore is avoided in high risk patients and those poor grade SAH patients from a ruptured aneurysm, due to brain retraction, difficulty of vessel dissection, and the need for longer anesthesia. There are usually greater hemodynamic changes during microsurgical clipping and brain manipulation compared with endovascular treatment. Postoperative cerebral blood flow and cerebral metabolic rate for oxygen may be decreased in the postoperative period secondary to brain retraction (26, 27).

Surgical trauma suffered during a craniotomy may not be well tolerated by older patients. Subgroup analysis of patients older than 65 years in the ISAT cohort showed that rates of epilepsy, infections, and pulmonary complications were higher in the clipping group than the endovascularly treated group (28).

The surgical approach is often limited in providing a 360° view of the aneurysm and associated perforators. In complex aneurysms part of the aneurysm neck may be hidden from view, resulting in residual aneurysm filling after the aneurysm neck is clipped (22). Aneurysms in the vertebrobasilar system are less accessible than those in the anterior circulation and sometimes require extensive cranial base approaches that are invasive and associated with high morbidity (21). This is especially true for posterior cerebral artery P2 segment, basilar trunk, proximal anteroinferior cerebellar artery, vertebrobasilar junction, and vertebral artery (VA) aneurysms where endovascular therapy is preferred (29).

In cases of giant aneurysms, size and morphology can create problems with surgery when a sufficiently large clip is not available to accommodate the aneurysm. Surgical morbidity and mortality from clipping of giant aneurysms has remained between 20 and 30% with specialized centers performing bypass and parent artery occlusion or aneurysm trapping for a large number of these aneurysms. Issues faced with these giant aneurysms are wide aneurysm necks, complex arterial branches, intraluminal thrombus, dysplastic vessel walls, and perforator vessels (30).

Conversely, blister aneurysms<3 mm can be challenging when a sufficiently small clip is unavailable to circumvent the aneurysm without compromising the parent vessel. They do not have well defined necks and have fragile walls making it difficult to clip primarily (31).

Successful clipping requires a pliable aneurysm neck to allow the clip blades to close. Some aneurysms have a highly calcified neck that precludes the closure of the aneurysm clip blades and hence the aneurysm continues to fill even after clip placement. Risk of distal embolization is also higher in these cases.

Repeat surgery for aneurysms that develop in a patient with a prior craniotomy are often challenging due to scar tissue, cerebral spinal fluid (CSF) leaks, and previously placed clips that prevent a clear view of the aneurysm site.

Aneurysms embedded in brain tissue, such as the brain stem, can be challenging to treat surgically as they involve dissection and possible injury to adjacent brain tissue during the clipping process (19).

## Limitations of Endovascular Coiling

Successful endovascular coiling depends on adequate packing of the aneurysm dome and base with coils. A high dome to neck ratio is crucial to retaining coils inside the aneurysm. A narrow neck allows primary coiling of the aneurysm with resultant aneurysm thrombosis and protection. A wide neck can preclude tight packing of coiling and is associated with a high risk of coil prolapse into the parent vessel.

In complex wide-based aneurysm morphologies a number of adjuncts may be used to promote a stable coil construct. This includes the use of compliant balloons to help remodel the coil construct to prevent coil prolapse (32). Similarly, stent-assisted coiling may be employed that uses stent lattice to bridge the aneurysm neck and prevents coil prolapse. Use of stent-assisted coiling requires anti-platelet agents, and thus cannot be safely used in most ruptured aneurysm situations (33). Double microcatheter techniques can sometimes be used to achieve stable coil constructs in multi-lobed complex aneurysms by covering recesses with coils from two separate microcatheters (34). More recently flow diverters, such as pipeline and silk devices, have been used to treat wide-based complex aneurysms in the anterior circulation. These flow diverters allow vessel wall reconstruction and neointimal growth across the aneurysm neck with aneurysm exclusion over the ensuing months secondary to flow stasis inside the aneurysm. However, a rigorous dual anti-platelet regimen is employed in pipeline patients until the aneurysm is excluded (35). A wide-neck aneurysm with complex morphology can make complete aneurysm exclusion with endovascular therapy alone difficult.

While newer tools and devices are available that allow endovascular treatment of many complex aneurysms, there is still a subset of aneurysms where combined endovascular and microsurgical management is recommended. Endovascular coiling can be challenging in patients with tortuous vascular anatomy that makes access to the aneurysm difficult. Tortuosity in the aortic arch and neck can interfere with placement of the guide catheter, precluding safe delivery of the microcatheter to the aneurysm site. Thus, a complete coiling of the aneurysm is often not possible. Small parent vessels can sometimes pose a challenge to safe wiring and microcatheterization.

Endovascular coiling of aneurysms with important vessels that originate from the aneurysm dome or base may not be performed safely without compromising flow. In such cases a partial coiling may be completed. While stent-assisted coiling and flow diverters may be used, they may pose an added risk to the patient treated with anti-platelet agents. In such a scenario surgery offers the option of clip reconstruction of the aneurysm while preserving important perforators and bifurcation vessels.

Management of pseudoaneurysms with endovascular coiling is highly challenging due to the risk of increased vessel perforation with the placement of coils into the subarachnoid space. In such scenarios endovascular sacrifice of the parent vessel may be necessary, otherwise microsurgical trapping of the aneurysm is required. Similarly mycotic and traumatic distal aneurysms may not be approached safely with endovascular therapy.

Coil mass extrusion is commonly observed when previously coiled patients are taken to the operating room. It could be misdiagnosed as compaction on angiography and is thought be a delayed process. Waldron et al. hypothesized this to be due to aneurysm perforation with resultant extrusion of coils, initial coiling of a pseudoaneurysm, coil compaction leading to extrusion, or breakdown of distal aneurysm wall (36).

## Endovascular Therapy after Surgical Treatment

Endovascular therapy after surgery is employed in a number of different scenarios. This could be as a result of primary failure of surgical therapy or in a planned fashion before any therapy is completed. The best decision regarding a combined strategy requires a diagnostic cerebral angiogram with 3D reconstructions. This provides the best understanding of the aneurysm shape, morphology, relationship to important branches, and the parent vessel.

### Coil embolization after partial aneurysm clipping

A number of aneurysms are treated with clipping given their wide-based morphology making stable coil constructs difficult. Microsurgical clipping allows protection of a large part of the aneurysm but, due to a limited surgical corridor and anatomical restraints, a part of the aneurysm may still continue to fill. In these situations microsurgical clip reconstruction of the aneurysm can help create a narrow neck, which makes subsequent coil embolization of the residual aneurysm possible. This scenario is encountered in carotid cave and paraclinoid aneurysms where part of the aneurysm extends beyond the dural ring. This unique anatomy prevents placement of a microsurgical clip without dissection of the cavernous sinus, which can have significant morbidity. Reconstruction of a narrow neck allows easy coiling of the aneurysm. While neck reconstruction to favor coiling can help, it can alter flow dynamics with a higher flow jet toward the aneurysm dome increasing likelihood of rupture in some cases. Therefore second stage coiling should be completed soon after clip reconstruction. Wrapping with muslin may afford protection from rupture during subsequent coiling.

A coil embolization may be used as bailout in aneurysms with a highly calcified neck. Due to the presence of atherosclerotic calcified plaques, closure of the aneurysm neck may not be complete. This leads to continued slow filling of the aneurysm through a narrow neck. A subsequent endovascular coil embolization of the aneurysm though the narrow calcified neck can help definitively thrombose the aneurysm.

Another common situation for coil embolization after clipping is with aneurysm recurrences. Patients with an enlarging aneurysm residual can be safely treated with coiling, possibly with balloon and stent assistance (37). This prevents the need for reoperation for a previously clipped aneurysm and avoids issues with scarring and high surgical risk.

Kim et al. demonstrated safety of coiling partially clipped aneurysms in their series of 24 patients with residual/recurrent aneurysms post clipping. The only predictor of poor outcome in these cases was presentation with re-rupture post clipping (38).

### Endovascular parent vessel occlusion after surgical bypass for an aneurysm

Some complex aneurysms may be closely associated with multiple vessels that make placement of a microsurgical clip impossible without compromising flows. Further, coiling may be challenging and coil configuration may not allow preservation of branch vessels. In these scenarios a microsurgical extracranial to intracranial bypass [superficial temporal artery (STA) to middle cerebral artery (MCA); external carotid artery (ECA) to MCA reverse saphenous vein or radial artery interposition graft] may be completed to provide adequate flow distal to the aneurysm. Once the bypass is in place via microsurgery an endovascular occlusion of the parent vessel feeding the aneurysm and the aneurysm itself can be completed. Performing an interval endovascular occlusion of the parent vessel obviates the need for additional operative surgery and may also allow time for the bypass to mature, if necessary. Additionally, a balloon test occlusion (BTO) can be performed prior to coil sacrifice of the vessel. The angiographic evaluation of the graft provides accurate assessment of graft patency prior to endovascular sacrifice.

Use of endovascular parent vessel occlusion (PVO) obviates the need for traditional, more invasive, routes of vessels sacrifice, such as surgical Hunterian ligation. Gaining surgical proximal control may require a neck scar and additional surgery to expose the cervical ICA.

Most endovascular arterial occlusions are performed within 24 h, and persist if successful at this time point. Staged PVO therapy allows assessment of bypass patency before sacrifice. Repeat BTO in a bypassed patient can confirm the adequacy of the bypass before sacrifice.

Sometimes a bypass can alter flows and cause flow stasis inside the aneurysm with no observable filling seen from the parent vessel. There is a real risk of aneurysm rupture after the bypass and before endovascular coil sacrifice. Therefore, an early endovascular sacrifice after bypass should be planned.

### Failure of surgical therapy

In rare scenarios when the surgeon determines that an aneurysm is unclippable, endovascular therapies may be employed. This may be due to operative visualization of perforators, an aneurysm embedded in eloquent brain parenchyma or failure of adequate proximal control. A previous clipping in a patient with multiple aneurysms may also block access, such as when a large clip or scarring obliterates adequate view.

## Surgical Treatment after Endovascular Therapy

Microsurgical clipping after initial endovascular treatment for the same aneurysm may be employed in a number of circumstances. These cases could be complete failure of coiling due to the limitations described earlier or could be an adjunctive therapy. Some of the therapies may be used concomitantly in the same setting.

### Clipping after coiling of aneurysm dome

Microsurgical clipping may be used as a more definitive therapy after initial coil embolization. This combined treatment is very useful in ruptured aneurysms where coiling provides short-term protection of the aneurysm dome until the patient recovers. Microsurgical clipping can then be completed in an interval fashion when better tolerated. Microsurgery in the setting of a ruptured aneurysm can be challenging due to hydrocephalus, brain edema, and altered cerebral hemodynamics. Coiling is thus better tolerated in the acute setting and with subsequent clipping if significant residual is present (36, 39). This situation may not be applicable to all aneurysms, especially blister and small aneurysms, which have a high risk of rupture with endovascular coiling. (40).

Civit et al. shared their early experience clipping ruptured aneurysms that were previously coiled in the acute stage. A good outcome was reported in this series that included partially coiled aneurysms, aneurysm recurrence, and/or re-rupture after coiling (41). In patients presenting with severe vasospasm, partial coiling followed by staged definitive clipping is a good algorithm to avoid microsurgical manipulation of vessels in the setting of vasospasm (42). Rabenstein et al. reported a high incidence of vasospasm in SAH patients treated with clipping versus coiling (43). The vasospasm treatment and coiling can be completed during the same procedure safely.

Waldron et al. reported their experience clipping previously coiled aneurysms. Partially coiled aneurysms are uncollapsible and difficult to manipulate. If coils extend to the aneurysm neck they can hold the aneurysm walls apart and transform the soft neck into a wedge that can splay clip blades, causing a clip to slide down the neck and occlude parent and branch vessels. Coils can prevent complete closure of the neck and can extrude into the subarachnoid space to complicate the dissection of the aneurysm or branch arteries. Thrombus formation can harden an aneurysm and sometimes coils may have to be removed before safe clipping can be performed. In some cases a bypass may be needed to safely remove coils and thrombus with clip reconstruction of the aneurysm (36). It is, however, advisable not to remove coils when possible.

### Preoperative BTO with clipping

Endovascular therapy is extremely useful in performing a preoperative balloon test occlusion of a proximal parent vessel prior to microsurgical vessel sacrifice. BTO is now widely used in patients with complex aneurysms that could require operative sacrifice of a parent vessel. This also helps determine which patients may require a bypass and whether a high or low flow bypass may be needed.

### Proximal control of aneurysm using temporary balloon occlusion

Appropriate proximal control of a vessel is a crucial tenet of microsurgical clipping. With the development of the neurosurgery microscope and improved drills, it is possible to drill down the anterior and posterior clinoid process for proximal ICA aneurysms and basilar apex aneurysms. This provides adequate proximal control but may be associated with additional morbidity and risks such as CSF leak from a pneumatized clinoid process. In other cases neck exposure may be required to provide proximal control through cervical ICA clamping. Cervical exposure of the ICA has additional risks of nerve injury and morbidity from the dissection itself. Batjer et al. first described the retrograde suction decompression technique for softening giant paraclinoid aneurysms. The technique required a cervical ICA exposure with an angiocatheter in the cervical internal carotid to provide suction decompression (44). Combined endovascular treatment can now be used to provide safe proximal control for both the ICA and VA. A balloon catheter may be advanced into the ICA/VA prior to aneurysm manipulation and inflated to obtain proximal occlusion. A dual lumen balloon catheter may then be used to create a suction decompression of the aneurysm, making it easier for the surgeon to manipulate the aneurysm. Newer compliant balloons have made the procedure low risk and easily tolerated (45).

Some advocate the use of smaller intracranial balloons to cause occlusion against the aneurysm neck and make clip placement easier (46).

### Proximal endovascular vessel occlusion prior to surgical debulking

Endovascular therapy can provide a safe avenue for PVO in patients with giant aneurysms who have passed a BTO. These patients may have mass effect from the aneurysm sac and proximal occlusion can make surgical debulking of the aneurysm easier.

## Stanford Experience

Over the past 13 years 67 cerebral aneurysms have been treated at Stanford University Medical Center (Table 2) by using combined treatment modalities. Each aneurysm was treated with a combination of endovascular and microsurgical techniques in either an early or interval fashion. Most of these aneurysms were ruptured complex aneurysms that were initially treated with endovascular coiling followed by microsurgical clipping. These cases highlight the paradigm that coiling provides initial short-term dome protection in patients who are unfavorable candidates for early surgery. These patients can then be brought back for more definitive clipping if a residual aneurysm is seen on follow-up imaging. Placement of a stent for assisted coiling does not preclude subsequent clipping, as we have found temporary occlusion of stented arteries is well tolerated, with the stents re-expanding to their initial size with removal of the temporary clips. Additionally, interval treatment allows use of adjunctive endovascular techniques, such as stent-assisted coiling and flow diverting devices.

**Table 2:** **Stanford neurosurgery experience with combined endovascular and microsurgical management of complex aneurysms (2000–2013)**.

**Total patients**	63	
**Gender**	20 Males (32%)	
	43 Females (68%)	
**Total aneurysms**	67	
**Ruptured**	53 (79%)	

**Aneurysm location (*n*)**	**Treatment**	
	**Microsurgery followed by endovascular (clip-coil) (rebled)**	**Endovascular followed by microsurgery (coil-clip) (rebled)**

ICA (9)	7	3 (1)
MCA (7)	2	5
Acomm and ACA (21)	2 (1)	19 (1)
Pcomm and anterior choroidal (12)	2	10 (1)
Basilar, PCA, VBJ (13)	1	12 (1)
PICA, vertebral (4)	0	4
Rebled before second treatment	1	4
Total cases	14	53

*Acomm, anterior communicating artery; ACA, anterior cerebral artery; ICA, internal carotid artery; MCA, middle cerebral artery; PCA, posterior cerebral artery, PICA, posterior inferior cerebellar artery; PComm, posterior communicating artery; VBJ, vertebrobasilar junction*.

The patients treated with endovascular techniques after microsurgery include a large number of ICA aneurysms. ICA aneurysms located proximally, such as paraclinoid and carotid cave aneurysms, can be challenging to completely obliterate with microsurgery. As highlighted above, extensive clinoidal drilling and exposure may be required (45). However, a combined approach can reconstruct the neck of some of these proximal ICA aneurysms making subsequent coiling possible.

A small subset of treated patients presented with a hemorrhage from the same aneurysm following their first treatment and was treated with the second modality. A total of three coiled patients bled from the treated aneurysms before being clipped. These coils did not completely obliterate the aneurysm base and bled as no management was done to secure the residual filling base. We therefore closely follow patients coiled in the acute setting to document any coil compaction and residual filling that could warrant additional treatment.

All patients in our series had complete aneurysm obliteration with the combination of therapies used and there was no procedural mortality from combined treatments.

There have been a number of clinical series since we shared our early clinical experience many years ago. We discussed our limited experience at that time with neck reconstruction using clipping followed by coiling (19, 22). Similarly, advantages of coiling aneurysms to reduce short-term bleeding risk in poor grade patients and aneurysms in difficult locations can allow more controlled and definitive clipping in an interval fashion. Over the years the rapid development of improved endovascular techniques has helped provide more definitive results with acute coiling. This includes use of balloon reconstruction techniques, development of more trackable microcatheters, and softer coils.

Hacein-Bay et al. treated a series of 12 patients with complex aneurysms using a combination of endovascular and microsurgical techniques. They achieved a 92% obliteration rate (18). They included patients who underwent exploratory aneurysm surgery without aneurysm clipping. Aneurysms assessed using cerebral angiography before clipping were also included under the combined management umbrella. We believe that all complex aneurysms should undergo 3D reconstructed image analysis to accurately assess branch vessel anatomy, as well as access to the aneurysm neck and surgical corridor. Management of complex aneurysms is thus inherently combined and in many cases cerebral angiography is crucial. In a few cases at our center the aneurysm was explored surgically and when inadequate access to aneurysm neck was evident, the dome was wrapped with muslin and the patient was taken for endovascular coiling. In cases where difficulty with surgical access may not be realized until surgery, unnecessary microsurgical exploration may be avoided with angiographic dual volume reconstruction to show the relationship between the aneurysm and bony anatomy.

Lawton et al. discussed their combined management of 77 patients with complex aneurysms and nicely illustrated their experience with surgical revascularization after aneurysm and PVO (21). They achieved a 95% aneurysm obliteration rate and 23 patients underwent surgical revascularization. Microsurgical bypass is a useful tool to manage complex aneurysms after a BTO and microsurgical/endovascular vessel sacrifice. This is especially true because there is no endovascular substitute for revascularization surgery.

Hoh et al. published their experience at Massachusetts General Hospital with complex aneurysm treatment, namely fusiform aneurysms, dissecting pseudoaneurysms, complex wide-necked aneurysms, and bifurcation aneurysms (20). They shared their experience using combined microsurgical and endovascular techniques to achieve flow alteration when primary clipping and coiling was not applicable. The goal for many of these lesions is flow alteration rather than direct aneurysm obliteration, which may not be possible. Microsurgical trapping, PVO, and revascularization bypasses to treat these aneurysms was used with a morbidity of 6.3% and mortality of 10.4%. Understanding flow dynamics and anatomy are crucial when managing this subset of complex aneurysms.

## Four Illustrated Cases

### Case 1

**Figure 1:**
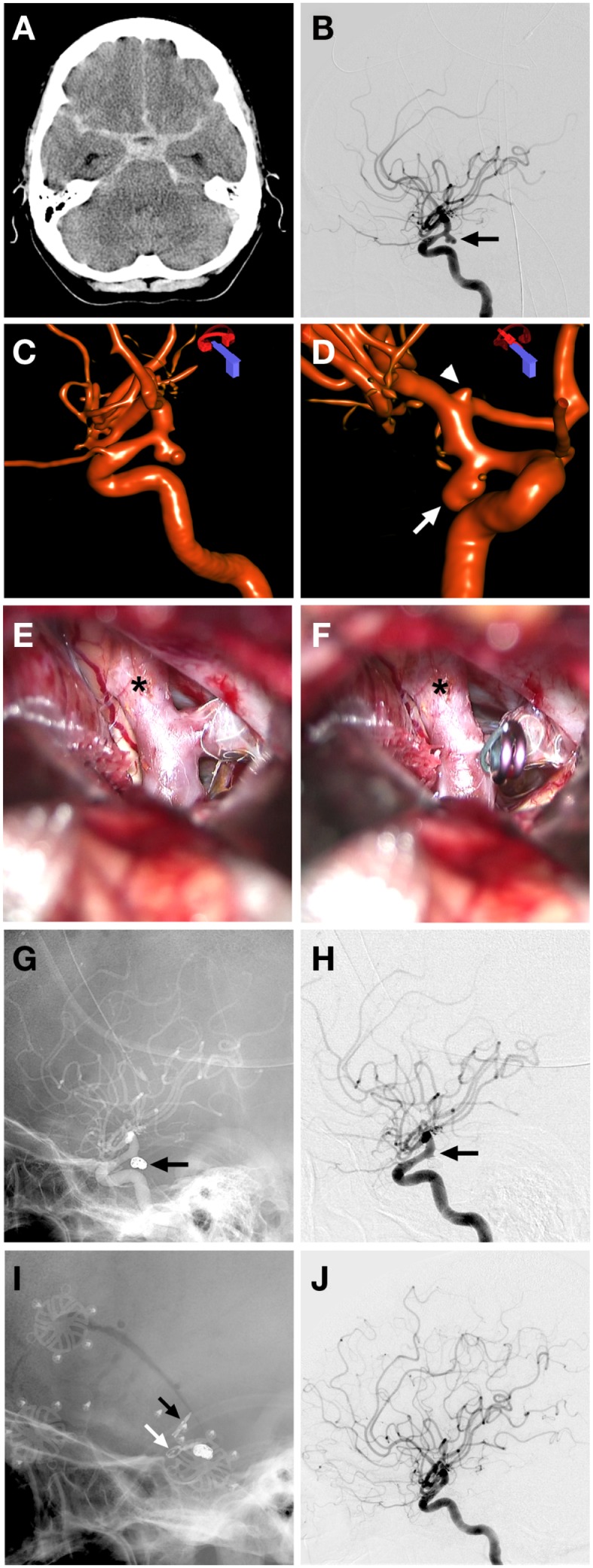
**Case 1**. **(A)** CT non-contrast demonstrating diffuse subarachnoid hemorrhage in the basal cisterns and prominent temporal horns; **(B)** lateral projection cerebral angiogram demonstrating a PComm aneurysm (arrow); **(C)** 3D angiographic reconstruction of right ICA showing the 5 mm Pcomm aneurysm; **(D)** another view of the 3D angiogram demonstrating the ruptured Pcomm aneurysm (arrow) and the 3 mm unruptured right ICA terminus aneurysm (arrowhead); **(E,F)** intraoperative microscope views showing exposure of the right Pcomm (asterisk), with visualization of coils in the aneurysm dome and continued filling of the aneurysm base **(E)** and obliteration of aneurysm residual after placement of aneurysm clip; **(G)** unsubtracted lateral cerebral angiogram demonstrating the coil mass obliterating the aneurysm dome (arrow); **(H)** subtracted lateral cerebral angiogram showing filling of residual aneurysm base (arrow); **(I)** magnified unsubtracted angiographic view post coiling and clipping with a microsurgical clip across the residual Pcomm aneurysm (white arrow) and ICA terminus aneurysm (black arrow); **(J)** final lateral subtracted angiogram showing complete obliteration of previously filling Pcomm aneurysm.

Combined endovascular-microsurgical management of a ruptured posterior communicating (PComm) aneurysm with vasospasm (Figure 1).

A 51-year-old female presented with a Hunt and Hess 3 (HH3), Fischer 3 SAH from a ruptured bi-lobed right PComm artery aneurysm. She presented 3 days after onset of headaches and was confused on presentation. An external ventricular drain was placed and the 5 mm PComm artery aneurysm was treated with endovascular coiling. She had vasospasm on presentation, which was treated with intra-arterial nicardipine. A small residual aneurysm remained after coiling. The patient continued to have severe vasospasm, which was treated with nicardipine and angioplasty on three subsequent occasions during hospitalization. She was also noted to have a small unruptured ICA bifurcation aneurysm on the right side. The patient made an excellent recovery and was discharged neurologically intact. A follow-up angiogram demonstrated a small residual aneurysm base in the previously coiled right PComm artery aneurysm, as well as an approximately 3 mm diameter right ICA terminus aneurysm. She was then taken to the operating room for clipping of the residual right PComm artery aneurysm and the unruptured ICA terminus aneurysm with complete obliteration of both.

### Case 2

**Figure 2:**
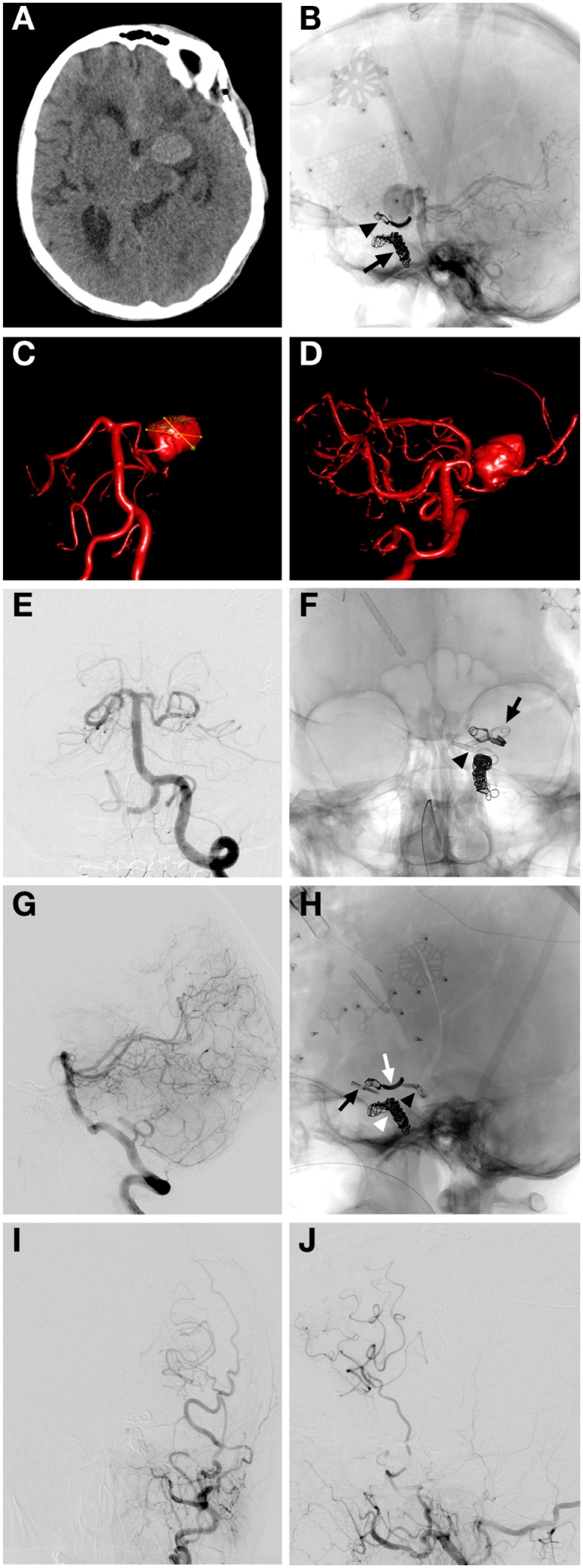
**Case 2**. **(A)** Non-contrast CT demonstrating a hyperattenuated left sylvian lesion with subarachnoid blood around the aneurysm and anterior interhemispheric fissure; **(B)** lateral unsubtracted skull radiograph showing the previously placed microsurgical clip (arrowhead) and coil mass from previous left ICA coil occlusion (arrow); **(C,D)** AP and oblique 3D angiographic reconstructions of vertebrobasilar system demonstrating the giant left supraclinoid ICA aneurysm being supplied by the left PComm; **(E)** AP subtracted angiographic view of left vertebral artery injection showing no filling of the giant aneurysm post clipping of left Pcomm; **(F)** AP skull x-ray demonstrating the two microsurgical clips used for feeding artery occlusions of a giant aneurysm with one clip over the Pcomm (arrowhead) and one over the supraclinoid ICA segment (arrow), previous coil mass from LICA occlusion and curved clip from previous clipping also seen; **(G)** lateral projection left vertebral artery angiographic injection showing no residual filling of giant aneurysm by the Pcomm; **(H)** lateral skull x-ray film demonstrating the clip from prior clipping (white arrow), coil mass from previous left ICA occlusion (white arrowhead), left Pcomm clip at the left Pcomm-P1 junction (black arrowhead), left supraclinoid ICA clip (black arrow); **(I,J)** AP and lateral left external carotid angiographic projections demonstrating filling of left superficial temporal artery with a patent bypass to left MCA and filling of left MCA territory post microsurgical occlusion of arterial feeders to aneurysm.

Coil sacrifice followed by microsurgical clipping of a recurrent giant ICA aneurysm (Figure 2).

A 62-year-old woman with a history of giant left supraclinoid ICA aneurysm underwent prior clipping of the aneurysm and subsequent coil sacrifice of the ICA at an outside hospital. Approximately 10 years after these treatments the patient was transferred to Stanford with an HH3, F3 SAH from the recurrent supraclinoid ICA giant aneurysm. The patient also had new onset of right facial droop with right hemiparesis and aphasia. A cerebral angiogram confirmed the presence of an approximately 2.6 cm diameter patent aneurysm involving the entire supraclinoid ICA with coil occlusion of the cavernous carotid artery and filling of the aneurysm from the left PComm artery from the vertebrobasilar injection. There was also some filling of the aneurysm through a reconstituted left ophthalmic artery. Injection of the right ICA revealed cross-filling to the left anterior and MCA across the anterior communicating artery (AComm). The patient had an external ventricular drain placed at the outside hospital. She underwent microsurgical clipping of the left PComm and supraclinoid ICA after an STA-MCA bypass to restore flow to the left hemisphere. She was then discharged to the transferring hospital after having a ventriculoperitoneal shunt placed.

### Case 3

**Figure 3:**
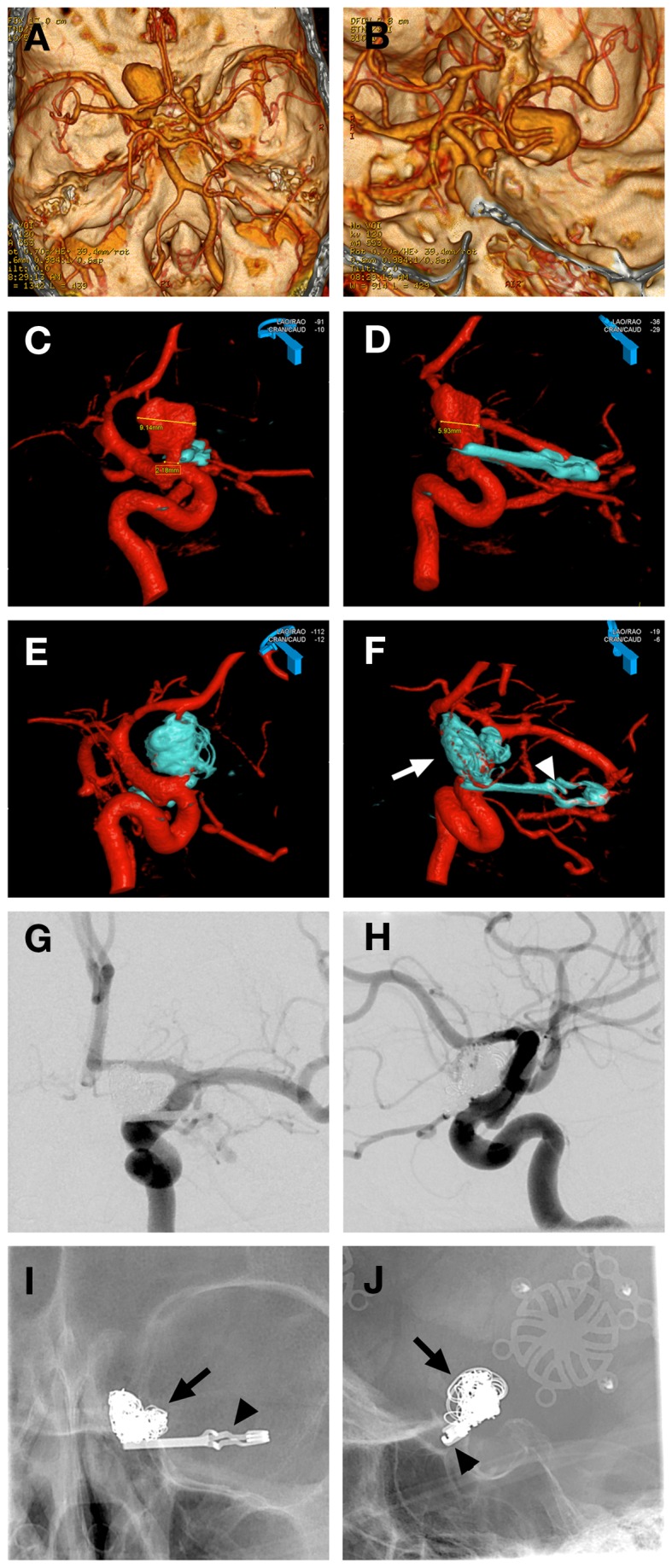
**Case 3**. **(A,B)** CT angiographic 3D reconstruction views showing the left giant carotid ophthalmic artery aneurysm; **(C,D)** 3D angiographic reconstructions from left ICA showing the left carotid ophthalmic artery aneurysm partially obliterated post clipping with a reconstructed narrow neck by the microsurgical clip (blue); **(E,F)** 3D angiographic reconstructions from left ICA post clipping and coiling with the giant aneurysm completely obliterated by the microsurgical clip (arrowhead) and the coil mass (arrow); **(G,H)** AP and lateral magnified subtracted left ICA angiographic views showing complete obliteration of aneurysm with clip and coils, ophthalmic artery is patent and well visualized **(H)**; **(I,J)** AP and lateral magnified skull x-rays showing the microsurgical clip (arrowhead) and coil mass (arrow).

Coiling of a residual giant carotid ophthalmic aneurysm following neck reconstruction after clipping (Figure 3).

A 66-year-old female presented to Stanford with worsening vision and was found to have a giant left carotid ophthalmic artery aneurysm. She was taken to the operating room for microsurgical clipping and developed a postoperative subdural hematoma and SAH from the residual aneurysm. An endovascular coil embolization of the residual aneurysm was completed with complete aneurysm obliteration. The initial microsurgical clipping remodeled the aneurysm neck making it narrow and more amenable to placement of platinum coils. The patient made a complete recovery and her vision improved.

### Case 4

**Figure 4:**
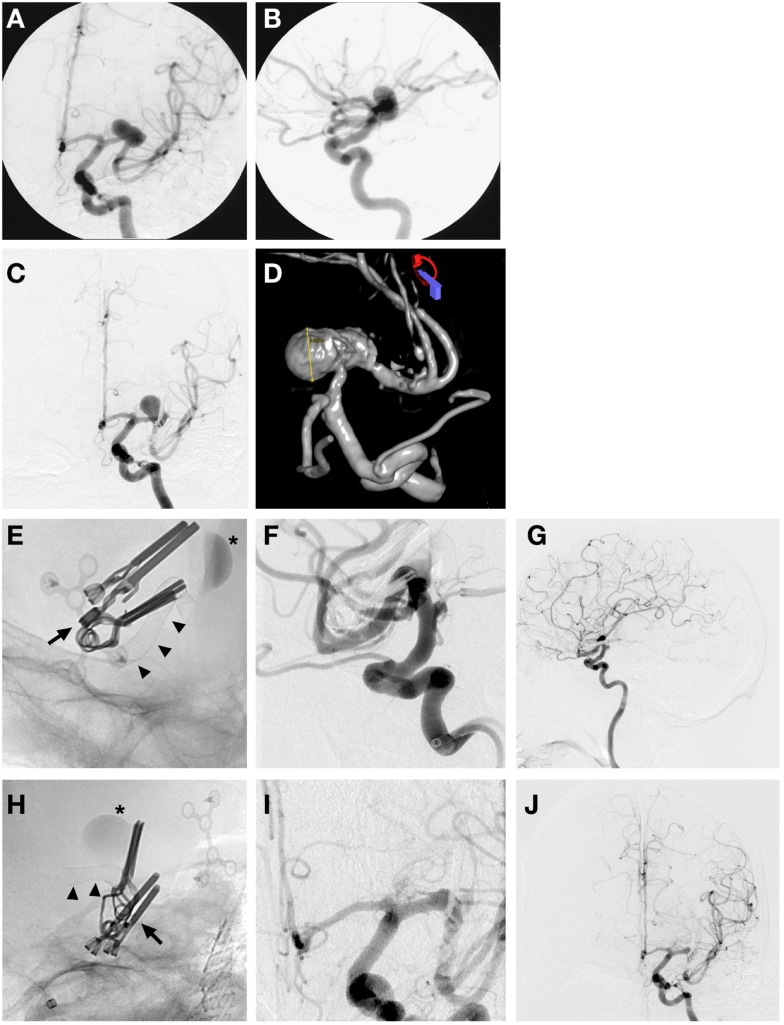
**Case 4**. **(A)** AP left ICA angiographic image demonstrating a wide-based large left MCA M1 segment aneurysm; **(B)** left MCA aneurysm seen on left ICA lateral angiographic projection; **(C)** AP left ICA angiographic image showing partial obliteration of aneurysm post clipping with enlargement and continued filling of a portion of the aneurysm dome 3 years after initial clipping; **(D)** 3D left ICA angiographic reconstruction post clipping showing the residual enlarging left M1 aneurysm; **(E,H)** lateral and AP magnified skull x-ray images immediately after placement of a Pipeline™ Embolization Device (Chestnut Medical Technologies, Menlo Park, CA, USA) (arrowheads) across the left MCA, clips from previous microsurgical clipping in place (arrow), contrast stasis in left MCA aneurysm indicating ongoing thrombosis; **(F,I)** magnified lateral and AP left ICA interval subtracted angiogram showing completely reconstructed left MCA with no residual aneurysm filling and patency of all M2 branches; **(G,J)** lateral and AP left ICA interval global angiographic images showing no residual left MCA aneurysm with a reconstructed left MCA and good filling of distal MCA circulation.

Endovascular placement of a pipeline embolization device after partial clipping of a ruptured MCA aneurysm (Figure 4).

A 68-year-old male presented with the worst headache of his life, and associated nausea and unsteadiness. Lumbar puncture demonstrated xanthochromia consistent with SAH. A CT angiogram and digital subtraction angiography confirmed the presence of a large 18 mm wide-based and partly fusiform aneurysm of the left M1 MCA prior to the left MCA bifurcation. The patient underwent a craniotomy with clip reconstruction and a STA-MCA bypass. He was noted to have a small residual aneurysm after clipping that increased in size on subsequent angiograms in the ensuing 3 years. He was then brought back for endovascular placement of a pipeline flow diversion device to treat his aneurysm. A follow-up angiogram 6 months later demonstrated complete obliteration of the aneurysm and a reconstructed MCA. This case illustrates how recent improvements in endovascular technology can be employed to successfully treat aneurysm recurrences after clipping.

## Future Directions

The authors recognize microsurgical and endovascular therapies as complementary, rather than competing, techniques for managing complex aneurysms. Improved surgical navigation systems, neurosurgical microscopes, microsurgery instrumentation, intraoperative ICG angiography, and intraoperative blood flowmeters promote safe microsurgical clipping of aneurysms. In parallel, technological advancements in endovascular neurosurgery have brought the use of balloons, catheters, and stents, which effectively treat aneurysms with complex morphologies. All complex aneurysms should ideally be evaluated after a diagnostic cerebral angiogram with 3D reconstructions. Hybrid operative suites combine access to excellent intraoperative angiography with availability of endovascular techniques, such as proximal balloon occlusion during the clipping process. Coil embolization of a parent vessel may also be completed in tandem with surgical revascularization in these hybrid suites (47).

While it is beneficial to employ combined microsurgical and endovascular therapy for many complex aneurysms, it is important to realize the technical difficulties when surgical therapy follows initial endovascular treatment. For example, scarring may interfere with clipping a partially coiled aneurysm, and placing clips across the neck is more difficult if coils have prolapsed into the base, preventing complete clip closure. Additionally, removing coils with surgical instruments to accommodate the clip may be hazardous. Similarly, coiling a previously clipped aneurysm can be difficult should the clip obscure normal working projections. Issues surrounding anti-platelet therapy and heparinization need to be emphasized and microsurgical therapy must be appropriately timed with endovascular therapy.

It is imperative for cerebrovascular surgeons in training to have a good grasp of microsurgical and endovascular technologies. Dual-trained neurosurgeons can safely combine treatments when beneficial, as well as understand the limitations of each treatment modality. Alexander et al. demonstrated that a combined endovascular and microsurgical aneurysm practice can offer equally good outcomes in both arms (48).

## Funding

This study was supported in part by Russell and Elizabeth Siegelman, Bernard and Ronni Lacroute, and the William Randolph Hearst Foundation (Gary K. Steinberg).

## Conflict of Interest Statement

The authors declare that the research was conducted in the absence of any commercial or financial relationships that could be construed as a potential conflict of interest.

## Appendix

### Key concept 1

Cerebral aneurysms can be complex due to a combination of factors relating to aneurysm anatomy, as well as the patient’s clinical condition.

### Key concept 2

Cerebral angiography with 3D reconstructions is crucial in planning aneurysm therapy for complex aneurysms, and a multidisciplinary team approach is necessary.

### Key concept 3

Coiling has low procedural morbidity and can be used for short-term dome protection of an aneurysm with an interval microsurgical clipping to obliterate any residual aneurysm.

### Key concept 4

Endovascular therapy of previously clipped residual aneurysms can be made easier by a narrow neck reconstruction during the clipping process.

### Key concept 5

Microsurgical revascularization bypass is a useful tool that can be employed for treatment of complex aneurysms after endovascular BTO and parent artery coil sacrifice.

### Key concept 6

A hybrid angiography-equipped operative suite is crucial to facilitate combined endovascular and microsurgical treatment of complex aneurysms in the future.
